# Reviewed article: Correia JVP, Silva-Oliveira F, Ferreira RC, Zarzar PMPA. Characterization, frequencies and comparison of child abuse reporting rates: a comparative study, Brazil, 2011-2021. Epidemiol Serv Saude. 2025:34;e20240545

**DOI:** 10.1590/S2237-96222025v34e20240545.a

**Published:** 2025-10-10

**Authors:** José Fernando Salvador Carrillo

**Affiliations:** 1 Instituto Federal de Alagoas, Macéio, AL, Brazil Instituto Federal de Alagoas Macéio AL Brazil

## Peer review A

## Questionnaire

Does the manuscript contain new and significant information to justify publication? Yes

Does the Abstract (Summary) clearly and accurately describe the content of the article? Yes

Is the problem significant and concisely stated? Yes

Are the methods described comprehensively? Yes

Are the interpretations and conclusions justified by the results? Yes

Is adequate reference made to other work in the field? Yes

## Manuscript Structure

Length of article is: Adequate

Number of tables is: Too many

Number of figures is: Adequate

Please state any conflict(s) of interest that you have in relation to the review of this paper. None

Interest: Good

Quality: Below Average

Originality: Average

Overall: Average

Recommendation: Major Revison

## Comments

Comentários Gerais: O trabalho apresentado pelos autores é relevante e aborda um tema de grande importância. No entanto, para fortalecer a clareza e a qualidade científica do estudo, seriam necessários alguns ajustes.

Similaridade do texto: O texto apresenta uma similaridade superior a 30% no relatório do Turnitin, sendo que a maior parte dos trechos correspondentes (21%) se refere a uma dissertação de mestrado. Solicitamos, por favor, que seja verificado se as paráfrases e citações estão em conformidade com as normas da revista.

Título: O título deve seguir as recomendações do STROBE. Recomenda-se que os autores o modifiquem para torná-lo mais conciso, claro e informativo. A palavra “perfil” gera ambiguidade, e o termo “estudo transversal” poderia ser colocado após os “:”, como no exemplo: “Violência infantil...: um estudo transversal”.

Gráfico e tabelas: Os títulos das ilustrações devem ser claros, informativos e autossuficientes.

Observa-se uma inconsistência entre os títulos das tables/gráfico o manuscrito. Enquanto os títulos de todas as tabelas e figuras indicam que os dados correspondem ao período de 2011 a 2022, as seções anteriores descrevem o período de 2011 a 2021.

As tabelas apresentam uma distribuição e organização dos dados que podem ser melhoradas. Sugere-se que os autores verifiquem a necessidade de incluir todas as variáveis, mesmo as de baixa frequência. Por exemplo, na variável “local da ocorrência”, há categorias com frequência inferior a 1% do total (“habitação coletiva”, “bar ou similar”, “local de prática esportiva” e “indústrias e construção”), sem que esses dados sejam discutidos no manuscrito. Uma alternativa seria agrupar essas categorias de baixa frequência sob a denominação “outros”.

Na Tabela 1, a ordem das variáveis também poderia ser ajustada. A variável “local da ocorrência” aparece primeiro, enquanto na seção de resultados, o texto começa com os tipos de violência. Recomenda-se que a sequência na tabela siga a ordem apresentada na seção de resultados para maior coerência.

Além disso, na Tabela 1, a percentagem de não resposta da variável “tipo de violência” (1,28%) não está clara, pois os critérios de seleção descrevem que notificações sem essa informação foram excluídas. Ainda, os valores e percentuais não coincidem. Por exemplo, o total de notificações para “negligência/abandono” é de 204.467, representando 53,5% dos casos da variável “tipo de violência”. No entanto, esse valor (n=204.467) não representa 53,5% do total de notificações (n=401.573) ou do total de casos após a exclusão de não respostas (396.433, equivalente a 100% - 1,28%). Sugere-se esclarecer a metodologia de cálculo.

Outro ponto a considerar é que as Tabelas 3 e 4 e o Gráfico 1 não são mencionados no texto, o que dificulta ao leitor identificar os dados a que se referem.

As Tabelas 3 e 4 são extensas. Recomenda-se avaliar a possibilidade de apresentar esses dados através de gráficos, como um gráfico semelhante ao Gráfico 1 ou por meio de um heatmap, para melhor visualização.

Finalmente, o gráfico 1 tem uma qualidade de resolução muito baixa.

Resumo : A conclusão do resumo não responde de forma adequada ao objetivo apresentado no próprio texto. Recomenda-se reformular a conclusão para que esta reflita claramente os principais achados e responda ao objetivo proposto.

Introdução: Nas linhas 39 e 40, o autor menciona que pelo menos um bilhão de crianças e adolescentes sofrem violência ou negligência. Seria recomendável delimitar temporalmente essa estimativa (por exemplo, especificar se se trata de “um bilhão de crianças por ano”).

Objetivo: O objetivo precisa ser reformulado para maior clareza e concisão.

O uso de inteligência artificial: Não foi identificado no texto se o autor utilizou inteligência artificial. Caso a revista solicite, recomenda-se que seja feita uma declaração sobre o uso, ou não, de softwares de inteligência artificial generativa e, se aplicável, uma descrição de como essa ferramenta foi empregada no processo de redação.

Métodos: Seria recomendável que a seção de métodos, bem como as demais seções do manuscrito, sigam as diretrizes do STROBE para estudos transversais.

Sugere-se também que os autores citem as fontes dos bancos de dados secundários utilizados nas análises (como SINAN, DATASUS, entre outros). Além disso, é importante que referenciem os documentos utilizados para estabelecer seus critérios na seção de métodos (por exemplo, o Instituto Brasileiro de Geografia e Estatística para a classificação de idade).

Para um leitor externo, o processo de notificação não está claro. Seria ideal que os autores detalhem como as notificações são realizadas, como as crianças são abordadas, e o contexto no qual as informações são coletadas.

É fundamental que os autores incluam a aprovação do comitê de ética para o estudo.

Além disso, recomenda-se que os autores descrevam o manejo dos aspectos éticos envolvidos, incluindo a anonimização (se aplicável), proteção e segurança dos dados.

Por fim, a metodologia para o cálculo das taxas não está clara. Sugere-se que os autores incluam a fórmula ou ajustem o texto para tornar o processo mais compreensível. 

Resultados: O processo de seleção dos casos ficaria mais claro com a inclusão de um diagrama de fluxo.

Recomenda-se que os autores aprimorem a redação da seção de resultados. Os parágrafos que descrevem as taxas de notificações por idade e sexo são extensos. Sugere-se priorizar os resultados mais relevantes do estudo para tornar a apresentação mais concisa.

Adicionalmente, os autores não incluíram dados numéricos para comparar as taxas. Seria útil justificar a decisão de omitir esses dados no texto.

Discussão: A discussão está bem estruturada, mas alguns aspectos podem ser aprimorados.

Na linha 24/25 da discussão, o autor compara seus resultados com “outras fontes de dados de violência no Brasil”, mas não cita a fonte específica.

Na linha 38, os autores mencionam “estudos nacionais (19,20) e internacionais (22,23)”, porém as ideias associadas a essas citações não foram desenvolvidas.

A Organização Mundial da Saúde é mencionada várias vezes ao longo do texto. Recomenda-se que os autores utilizem a sigla (OMS) nas menções subsequentes, para reduzir o tamanho do texto.

Por fim, sugere-se reduzir ou dividir algumas ideias em parágrafos mais curtos, pois atualmente alguns parágrafos estão extensos

